# The impact of two-dimensional and three-dimensional computed tomography on the decision to operate in distal radius fractures: a multicenter survey-based study

**DOI:** 10.1186/s13018-026-07049-y

**Published:** 2026-06-27

**Authors:** Osman Görkem Muratoğlu, Cem Yıldırım, Hasan Ceylan, Gökhan Güzel, Volkan Karaduman, Necati Doğan

**Affiliations:** 1 Department of Orthopedic and Traumatology, Atlas University Hospital, Barbaros Mah, H. Ahmet Yesevi Cad, No: 149. Güneşli, Bağcılar, Istanbul, Turkey; 2 Department of Orthopedic and Traumatology, Başakşehir Çam and Sakura City Hospital, Başakşehir Mahallesi G-434 Caddesi No: 2L, Başakşehir, Istanbul, Turkey; 3 Department of Orthopedics and Traumatology, Faculty of Medicine, Istanbul Yeni Yüzyıl University, Istanbul, Turkey; 4 Department of Orthopedics and Traumatology, Medicana International Beylikdüzü Hospital, Beylikdüzü Cd. No: 3, 34520 Istanbul, Turkey

**Keywords:** Distal radius fractures, Computed tomography, Three-dimensional imaging, Decision to operate, Survey study

## Abstract

**Objective:**

Imaging modalities play a critical role in determining surgical versus conservative management for distal radius fractures. This study aimed to evaluate the impact of two-dimensional (2D) and three-dimensional (3D) computed tomography (CT) on the decision to operate in distal radius fractures and to compare their influence between AO Type B and Type C fractures.

**Methods:**

This cross-sectional, survey-based study included 97 orthopedic and traumatology specialists. Twelve distal radius fracture cases classified according to the AO system (six Type B and six Type C) were selected. Participants were sequentially presented with plain radiographs, post-reduction radiographs in cast, 2D CT images, and 3D CT reconstructions for each case. After each imaging stage, participants were asked to indicate their decision to operate (surgical or conservative). Changes in the decision to operate were statistically analyzed.

**Results:**

Among AO Type B fractures, the addition of CT imaging to plain and post-reduction radiographs did not significantly change the decision to operate in most cases (*p* > 0.05). In contrast, among AO Type C fractures, the addition of 2D CT imaging significantly changed the decision to operate in favor of surgical management (*p* < 0.05), whereas the subsequent addition of 3D CT did not produce a further significant change (*p* > 0.05).

**Conclusion:**

For AO Type B distal radius fractures, the addition of CT imaging to plain and post-reduction radiographs had limited impact on the decision to operate. In AO Type C fractures, 2D CT imaging significantly influenced the decision to operate, whereas the subsequent addition of 3D CT did not provide an additional impact on the decision to operate.

**Level of evidence III:**

Descriptive survey study.

## Introduction

Distal radius fractures are among the most common upper extremity fractures in the adult population, accounting for approximately 15–20% of all fractures. They are frequently observed in the elderly population as a result of low-energy trauma associated with osteoporotic bone, whereas in younger patients they typically occur following high-energy trauma and often present as complex intra-articular fractures. The choice of treatment is critical for restoring wrist function, minimizing the risk of post-traumatic arthritis, and preserving overall quality of life [1, 2].

The decision to operate in distal radius fractures are based on several factors, including fracture stability, articular surface involvement, degree of displacement, extent of comminution, and patient-related factors such as age, functional demands, and comorbidities [3, 4].

Accurate assessment of these parameters largely depends on radiological imaging modalities. Standard posteroanterior and lateral wrist radiographs are considered the first-line imaging method for the evaluation of distal radius fractures. However, particularly in intra-articular fractures, plain radiographs may be insufficient to fully delineate fracture morphology and articular step-off. Therefore, computed tomography (CT) is widely utilized, especially in AO Type B and Type C fractures [5].

Two-dimensional CT slices provide valuable information regarding articular step-off and fracture comminution. In recent years, three-dimensional CT reconstructions have been increasingly incorporated into surgical planning, as they are thought to enhance understanding of fracture geometry [6]. Nevertheless, whether 3D CT truly provides additional value in determining the need for operative treatment and whether it offers superiority over 2D CT remain matters of debate.

The aim of the present study was to evaluate the impact of 2D and 3D CT imaging on the decision to operate in distal radius fractures, to compare their influence between AO Type B and Type C fractures, and to determine whether 3D CT leads to additional changes in the decision to operate compared with 2D CT.

## Materials and methods

### Study design

This study was designed as a cross-sectional, descriptive, survey-based investigation to evaluate the impact of different imaging modalities on the decision to operate in distal radius fractures. The study design allowed participants to sequentially assess different imaging stages of the same cases and to provide independent decisions to operate at each stage. This structure enabled a systematic evaluation of potential changes in the decision to operate across imaging modalities.

The study was conducted in accordance with established ethical standards for research involving human participants and adhered to the principles of the Declaration of Helsinki. All participants were informed in detail about the purpose and scope of the study. Participation was voluntary, and written informed consent was obtained from all participants. The study protocol was reviewed and approved by the relevant local ethics committee. Participant identities were anonymized, and all data were used exclusively for scientific purposes. The study included only orthopaedic surgeons. No patients or minors were involved. Therefore, parental consent was not applicable.

### Participants

A total of 97 orthopedic and traumatology specialists were included in the study. All participants were actively engaged in clinical practice and experienced in the diagnosis and management of distal radius fractures. Data regarding professional experience and institutional affiliation (university hospital, training and research hospital, public hospital, private hospital, etc.) were recorded to ensure a heterogeneous sample in terms of clinical background and practice setting.

Participants were not provided with any directive information during the study. At each imaging stage, they were asked to make independent decisions reflecting their own clinical judgment. Each participant evaluated all cases and all imaging stages.

### Case selection

Twelve distal radius fracture cases classified according to the AO/OTA Fracture and Dislocation Classification system were included in the study:

- Six AO Type B fractures (partial articular fractures).
- Six AO Type C fractures (complete articular fractures).

All cases were selected and classified according to the appropriate AO classification subtypes. To enhance the accuracy and reliability of the classification, all classifications were independently reviewed and confirmed by all authors. Initial fracture classification was performed by an experienced orthopedic surgeon familiar with the AO classification system.

Cases were selected from commonly encountered clinical scenarios that could create uncertainty in surgical indication and decision to operate. Open fractures, cases with associated neurovascular injury, or those with severe soft tissue damage were excluded to allow clearer assessment of the impact of imaging modality on decision to operate.

All cases were retrospectively selected from institutional archives. Patient identifiers were anonymized, and imaging data were standardized for presentation. No participants under the age of 18 were included in this study.

### Imaging protocol

All patients initially underwent standard radiographic evaluation at presentation. For displaced distal radius fractures, closed manual reduction was routinely performed in the emergency department, followed by post-reduction radiographs. Subsequently, CT imaging, including both two-dimensional (2D) and three-dimensional (3D) reconstructions, was obtained after reduction based on the post-reduction fracture alignment.

Each case was presented to participants in a standardized sequential order. The imaging stages were as follows:

Standard posteroanterior and lateral wrist radiographs.

Post-reduction radiographs obtained after cast immobilization.

Two-dimensional computed tomography (axial, sagittal, and coronal slices).

Three-dimensional computed tomography (rotational reconstructions from multiple angles).

(Dynamic images provided as GIF – Graphics Interchange Format)

(In the three-dimensional CT reconstructions, the carpal bones were not included; animated GIF images were created to display only the distal radius and distal ulna.)

Tomographic imaging refers to standard CT evaluation, whereas 2D CT and 3D CT were used to distinguish between planar and reconstructed volumetric assessments where applicable.

**Fig. 1:**
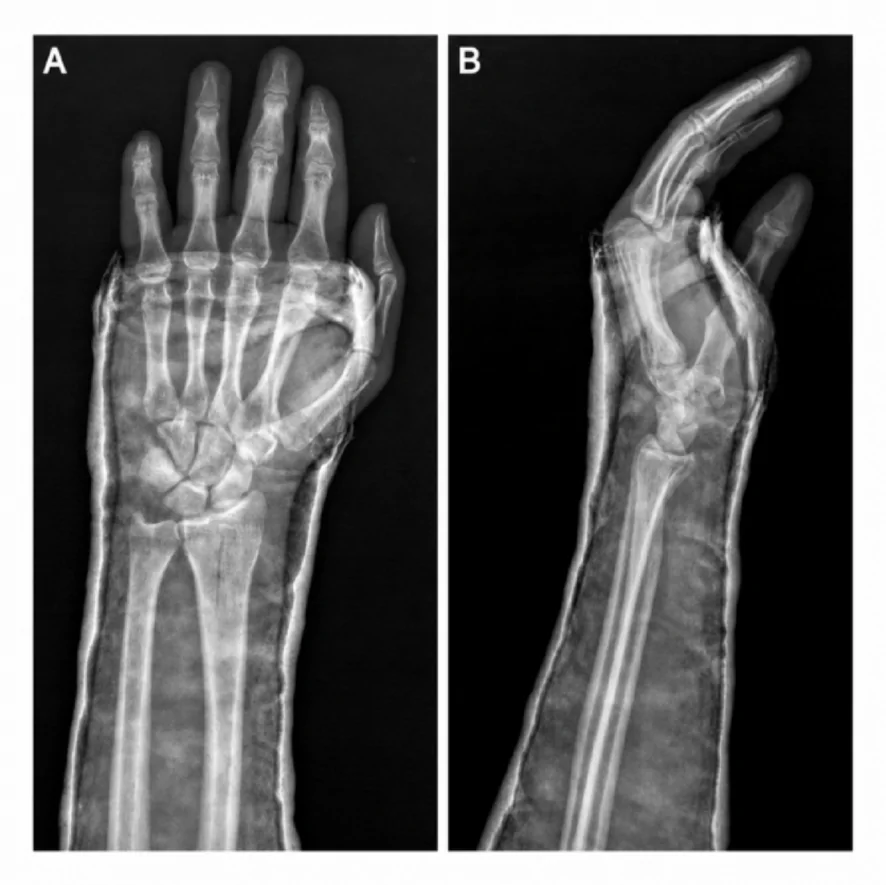
Post-reduction posteroanterior (**A**) and lateral (**B**) radiographs of an AO type B distal radius fracture following closed reduction and immobilization

**Fig. 2:**
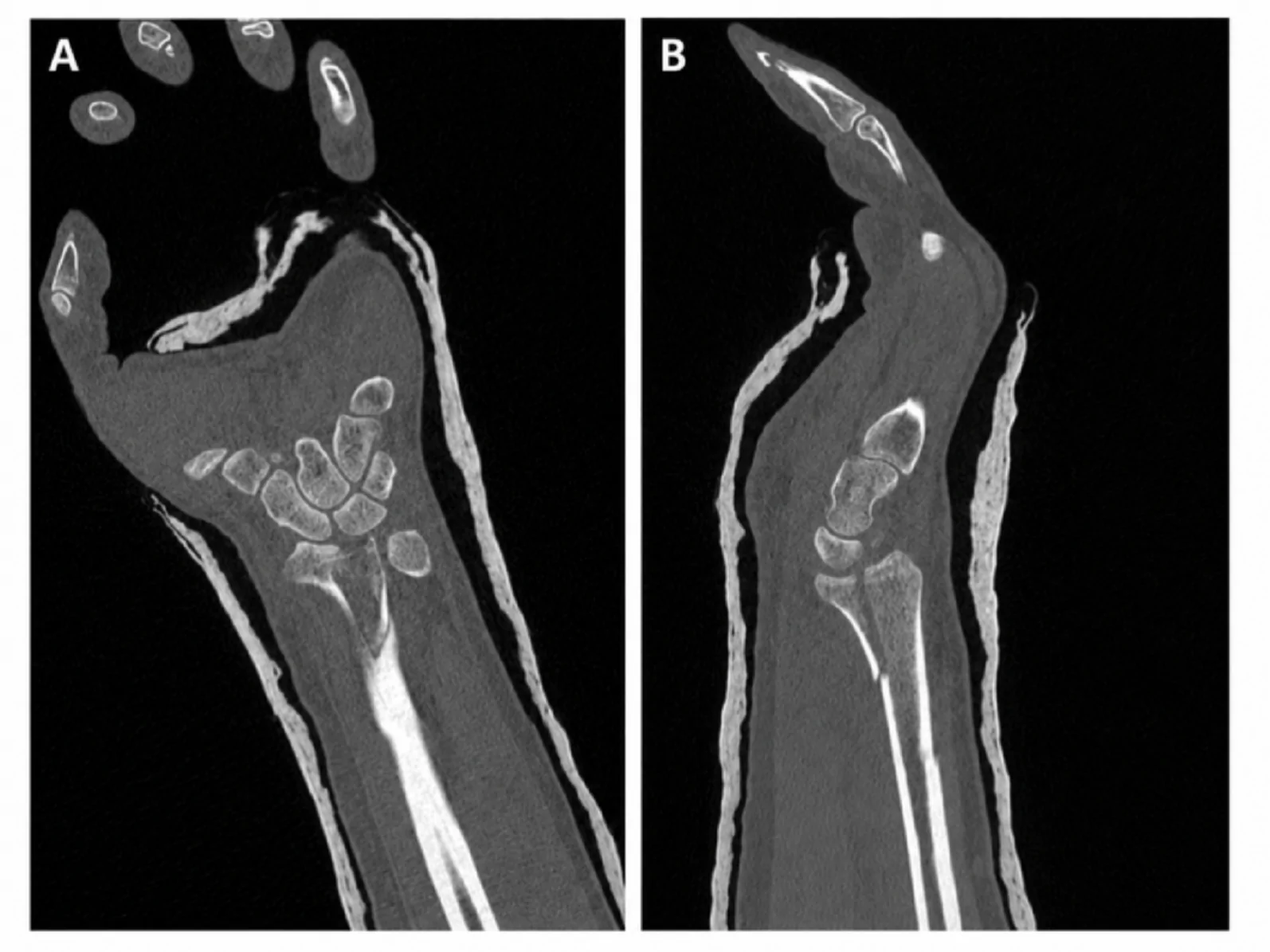
Representative 2D CT sections selected from the post-reduction GIF sequence of an AO Type B distal radius fracture.** A** Coronal reconstructed section.** B** Sagittal reconstructed section

**Fig. 3:**
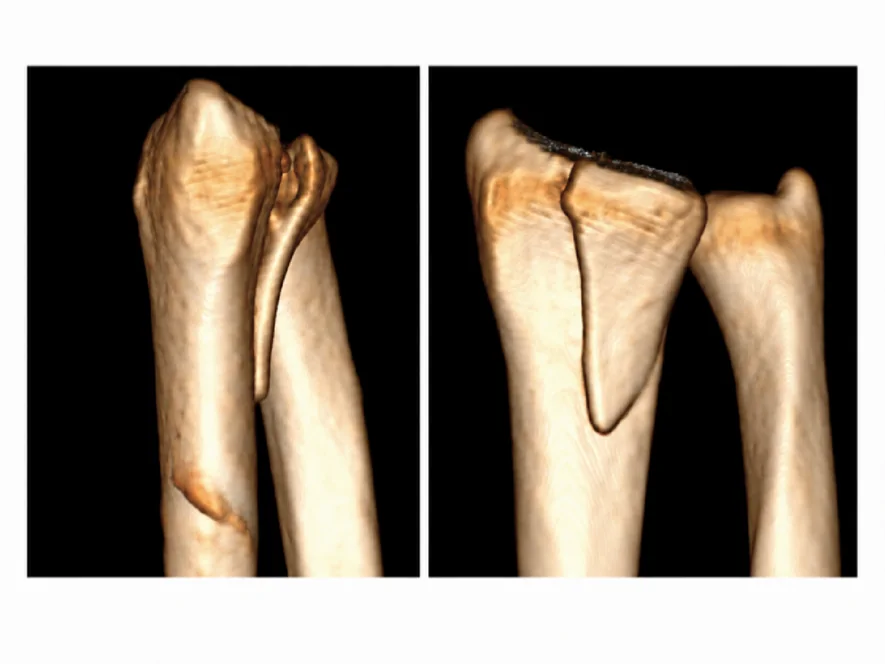
Representative 3D CT reconstruction images selected from the post-reduction GIF sequence of an AO Type B distal radius fracture, showing the spatial configuration of the fracture fragments after reduction

**Fig. 4:**
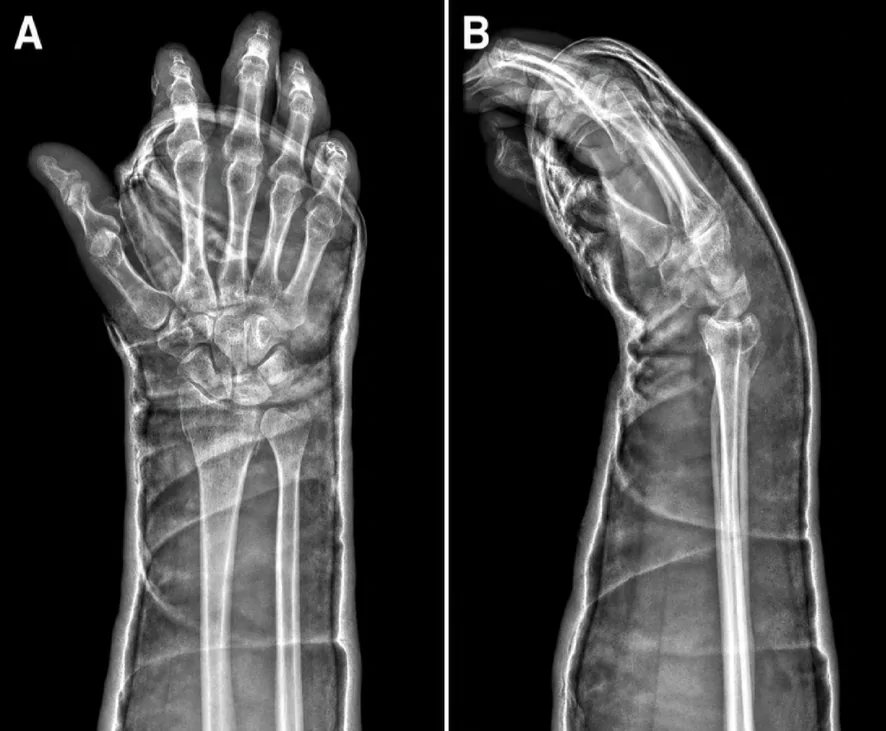
Post-reduction posteroanterior (**A**) and lateral (**B**) radiographs of an AO type C distal radius fracture following closed reduction and immobilization

**Fig. 5:**
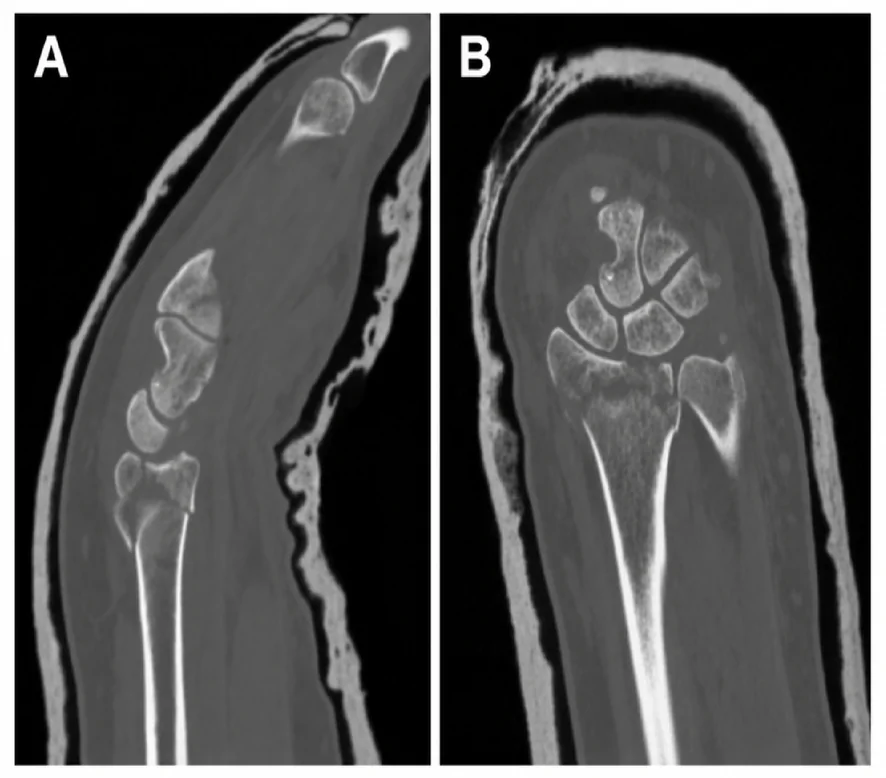
Representative 2D CT sections selected from the post-reduction GIF sequence of an AO Type C distal radius fracture.** A** Sagittal reconstructed section.** B** Coronal reconstructed section

**Fig. 6:**
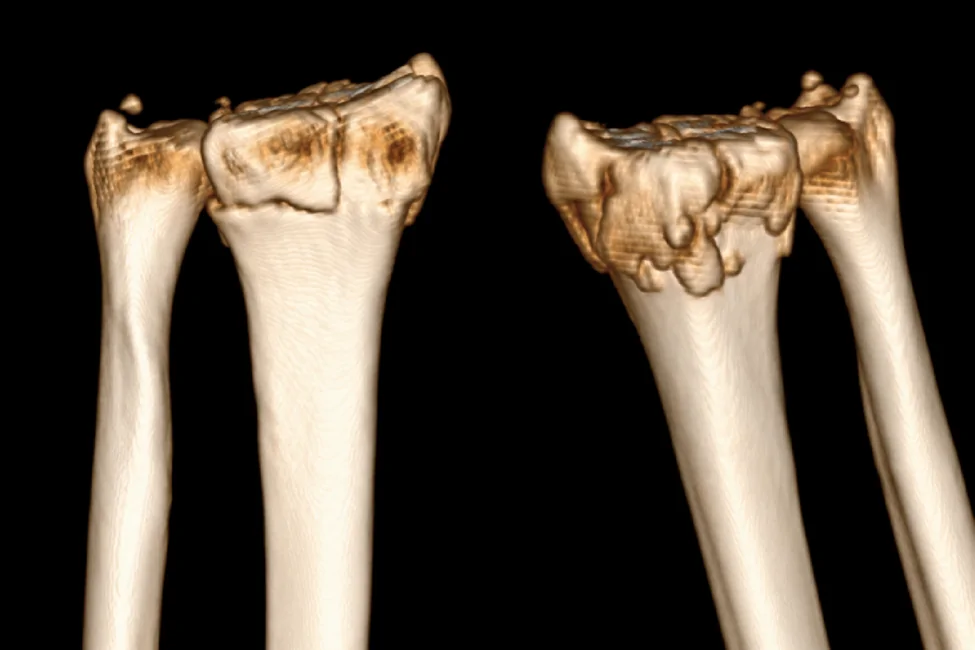
Representative 3D CT reconstruction images selected from the post-reduction GIF sequence of an AO type C distal radius fracture, showing the spatial configuration of the fracture fragments after reduction

The order of image presentation was identical for all participants. Each subsequent imaging stage was evaluated with access to the information provided by the previous imaging stages, thereby creating a cumulative imaging assessment process. Decisions to operate were recorded before proceeding to the next imaging stage. At each stage, participants were asked to select either “surgical” or “conservative” treatment (Figs. 1, 2, 3, 4, 5 and 6).

Participants did not have access to their previous responses and were asked to reassess each case solely based on the imaging data available at the current stage. This approach enabled objective analysis of changes in the decision to operate attributable to the imaging modality.

### Data collection and outcome measures

Data were collected electronically through a structured survey form. Each participant’s responses were recorded individually and coded on a case-by-case basis. Changes in decisions to operate across imaging stages for the same participant were analyzed in a paired manner.

Because the imaging modalities were presented in a fixed sequential order, the decision to operate at each stage were based on cumulative imaging information. Accordingly, the study design consisted of three cumulative imaging sets:

1. Plain radiographs and post-reduction radiographs.
2. Plain radiographs + post-reduction radiographs + 2D CT images.
3. Plain radiographs + post-reduction radiographs + 2D CT images + 3D CT reconstructions.

For convenience of expression throughout the manuscript and tables, these cumulative imaging sets are referred to as “CR + cast radiographs,” “2D CT,” and “3D CT,” respectively. Therefore, the terms “2D CT” and “3D CT” should be interpreted as incremental imaging evaluations rather than standalone independent imaging modalities.

The primary outcome measure was the rate of change in the decision to operate as imaging progressed (plain radiograph → post-reduction radiograph → 2D CT → 3D CT). Secondary analyses compared changes in the decision to operate between AO Type B and Type C fractures.

### Statistical analysis

All data were transferred to appropriate statistical software for analysis. Categorical variables were expressed as frequencies and percentages (%). Changes in the decision to operate across imaging modalities were analyzed comparatively. Appropriate nonparametric tests were used for the comparison of dependent categorical data. Specifically, McNemar’s test was used for paired categorical comparisons between imaging modalities. For pairwise comparisons between imaging modalities, McNemar’s test with Bonferroni correction was applied to control for multiple comparisons and reduce the risk of Type I error.

Differences in the decision to operate between AO Type B and Type C fractures were also analyzed separately. All statistical tests were two-tailed, and a* p*-value < 0.05 was considered statistically significant. Results were interpreted within a 95% confidence interval.

## Results

The distribution of participants according to years of professional experience is summarized in Table 1. Of the 97 participants, 22 had ≤ 5 years of experience, 36 had 5–10 years of experience, and 39 had ≥ 10 years of professional experience.

Among the six cases classified as AO Type B fractures, a statistically significant change in the decision to operate toward surgical management following 2D or 3D CT evaluation was observed in only one case. In the remaining five cases, advanced imaging modalities did not produce a statistically significant impact on the decision to operate (***p*** **> 0.05**).

Among the six AO Type C fracture cases, the addition of 2D CT imaging to plain and post-reduction radiographs resulted in a statistically significant change in the decision to operate in four cases (***p*** **< 0.05**). Although cumulative evaluation including 3D CT demonstrated significant differences in five cases, the subsequent addition of 3D CT reconstruction itself did not produce a further statistically significant change in the decision to operate.

When all cases were analyzed collectively, no statistically significant difference was found between cumulative evaluation including 2D CT and cumulative evaluation including 3D CT in terms of changes in the decision to operate. This finding suggests that 3D CT does not provide additional benefit over 2D CT in influencing the decision to operate.

Detailed distributions of decisions for surgical and conservative management for all cases, along with statistical comparisons, are presented in Table 2. (Tables 1 and 2).

**Table 1:** Comparison of decisions to operate for AO type B fractures according to imaging methods

	Treatment	CR + Cast	2D-CT	3D-CT	*p* value*	*p* value**	*p* value***
Case 1	Conservative	75	27	21	***0.001***	***0.001***	0.327
	Surgical	22	70	76			
Case 2	Conservative	25	29	30	0.255	0.428	0.876
	Surgical	72	68	67			
Case 3	Conservative	26	28	27	0.750	0.872	0.874
	Surgical	71	69	70			
Case 4	Conservative	84	88	87	0.367	0.507	0.810
	Surgical	13	9	10			
Case 5	Conservative	77	76	78	0.861	0.858	0.724
	Surgical	20	21	19			
Case 6	Conservative	70	68	68	0.752	0.752	1
	Surgical	27	29	29			

*Comparison between cumulative evaluation using CR + cast radiographs and cumulative evaluation including 2D CT

** Comparison between cumulative evaluation using CR + cast radiographs and cumulative evaluation including 3D CT

*** Comparison between cumulative evaluation including 2D CT and cumulative evaluation including 3D CT

**Table 2:** Comparison of decisions to operate for AO type C fractures according to imaging methods

	Treatment	CR + Cast	2D-CT	3D-CT	*p* value*	*p* value**	*p* value***
Case 7	Conservative	8	2	2	***0.026***	***0.026***	1
	Surgical	89	95	95			
Case 8	Conservative	34	28	29	0.358	0.445	0.875
	Surgical	63	69	68			
Case 9	Conservative	42	29	25	***0.026***	***0.005***	0.524
	Surgical	55	68	72			
Case 10	Conservative	20	11	13	***0.001***	0.182	0.664
	Surgical	77	86	84			
Case 11	Conservative	14	8	6	0.088	***0.029***	0.581
	Surgical	83	89	91			
Case 12	Conservative	62	26	20	***0.001***	***0.001***	0.171
	Surgical	35	71	77			

*Comparison between cumulative evaluation using CR + cast radiographs and cumulative evaluation including 2D CT

**Comparison between cumulative evaluation using CR + cast radiographs and cumulative evaluation including 3D CT

***Comparison between cumulative evaluation including 2D CT and cumulative evaluation including 3D CT

## Discussion

The principal finding of this study is that the impact of CT imaging on the decision to operate differs substantially between AO Type B and AO Type C distal radius fractures. In AO Type B fractures, the addition of CT imaging to plain and post-reduction radiographs rarely altered the decision to operate. In contrast, in AO Type C fractures, the addition of 2D CT significantly influenced the decision to operate, whereas the subsequent addition of 3D CT reconstruction did not provide a further significant impact on the decision to operate. These findings suggest that the primary contribution of CT in complex distal radius fractures is largely derived from conventional 2D cross-sectional imaging rather than from additional 3D reconstruction.

In contrast, for AO Type B fractures, plain radiographs appeared sufficient for determining whether surgery was indicated in the majority of cases. According to the AO/OTA Fracture and Dislocation Classification system, this group comprises partial articular fractures, in which articular involvement is limited and fragment complexity is generally lower. Accordingly, in our series, evaluators may have favored conservative treatment for AO Type B fractures when displacement was minimal and overall joint alignment appeared acceptable. As a result, fracture morphology can often be adequately assessed using standard posteroanterior and lateral radiographs. The relative ease of identifying articular step-off and displacement radiographically may explain why advanced imaging did not produce a significant change in the decision to operate in most Type B cases. In some cases, although plain radiographs suggested acceptable alignment and conservative treatment, CT images revealed more pronounced instability, displacement, and articular step-off, which may explain why surgeons shifted their decision toward operative management. This finding suggests that routine CT imaging in fractures with simpler morphology may not always alter management decisions.

Conversely, AO Type C fractures, characterized by complete articular involvement and frequent comminution, are often inadequately evaluated using two-dimensional radiographs alone. Metaphyseal comminution, dorsal or volar fragment configuration, and the degree of articular step-off may be underestimated on plain radiographs. The addition of 2D CT imaging more clearly demonstrated fragment displacement, metaphyseal comminution, and articular incongruity, thereby significantly influencing the decision to operate toward surgical management. In a study by Cole and colleagues, 2D CT was reported to provide a more accurate assessment of fracture displacement compared with conventional radiography [7]. Our findings are consistent with this observation and support the view that the primary diagnostic contribution of CT in AO Type C fractures is achieved through conventional 2D cross-sectional evaluation. Although AO-C fractures, particularly those with significant articular collapse, frequently require surgical treatment, initial closed reduction remains part of our institutional protocol for all displaced distal radius fractures. This approach may help restore alignment, alleviate pain, reduce soft-tissue tension, and provide temporary stabilization prior to definitive surgical management.

An important finding of this study is that the subsequent addition of 3D CT reconstruction did not produce a further significant change in the decision to operate beyond the information already obtained from 2D CT imaging. Three-dimensional reconstructions enhance visual comprehension of fracture geometry and improve spatial perception, particularly in complex articular configurations. Gryska and colleagues reported that 3D reconstructions facilitate understanding of fracture morphology [8]. However, in our study, the essential information required for determining surgical versus conservative management was largely obtainable from 2D cross-sectional images. This finding suggests that changes in the decision to operate are primarily driven by the objective visualization of displacement and articular incongruity provided by 2D CT imaging, whereas additional three-dimensional visualization may improve spatial understanding without substantially altering the decision to operate.

Previous studies have indicated that 3D CT may be beneficial primarily in preoperative planning, implant selection, and deformity analysis rather than in changing the initial decision to operate [9–11]. Three-dimensional imaging has been described as helpful in refining surgical approach and implant configuration. However, in most of these studies, the ability of 3D CT to change the fundamental treatment decision (surgical versus conservative) has not been clearly demonstrated. Our results support the notion that 3D CT may be more valuable in the detailed planning phase of surgery rather than in the initial determination of whether surgery is indicated.

In this study, visual assessment rather than quantitative radiological measurements was used. Considering that many clinical decisions in routine practice are based on rapid visual interpretation, this approach reflects real-world decision to operate. Nevertheless, as noted by O’Malley and colleagues, purely visual evaluation may not fully substitute for objective measurements [12]. To mitigate this limitation, cases were carefully selected, and those with poor image quality or potential for significant measurement ambiguity were excluded. Consequently, the selected cases were considered suitable for reliable visual assessment.

The wide range of professional experience among participating surgeons enhances the generalizability of our findings. Including orthopedists with varying levels of seniority and clinical backgrounds ensures that the results reflect general orthopedic practice rather than the perspective of a specific subgroup. This heterogeneity also provides indirect insight into how imaging modalities influence clinicians across different experience levels.

To address the lack of a unified quantitative surgical indication standard and potential inter-observer variability, all cases were presented in a standardized sequential format using identical imaging datasets. The aim of the study was not to define absolute surgical thresholds, but rather to evaluate how incremental imaging modalities influence individual surgeons’ decisions to operate, thereby allowing each participant to serve as their own control across imaging stages.

Despite its strengths, this study has several limitations. First, as a survey-based investigation, it was not supported by actual clinical outcomes, functional scores, or long-term follow-up data. Second, the relatively limited number of cases and reliance on static imaging prevented incorporation of additional clinical factors such as physical examination findings and patient expectations. The relatively limited number of cases included (six per AO fracture type) may restrict the generalizability of the study findings. The dichotomous classification of decisions to operate as surgical or conservative may oversimplify the multifactorial nature of clinical decision-making in distal radius fractures. Furthermore, the fixed imaging sequence may have introduced a potential sequence or learning effect. However, a change in the decision to operate does not inherently indicate a more accurate or superior clinical decision, as this study did not assess clinical follow-up, functional outcomes, or complication rates. Only* p*-values were reported, and effect size measures such as absolute differences or odds ratios were not included, which may limit the clinical interpretability of the results.

Notwithstanding these limitations, the relatively large number of participants and the standardized imaging protocol represent important strengths of the study. The fact that all participants evaluated the same cases in the same sequence enabled a clearer comparison of the impact of different imaging modalities on decision to operate. However, a direct comparison of independent imaging modalities would require randomized image presentation, which represents a limitation of the current study design. Although participant experience was documented to characterize the clinical background of the study cohort, no stratified analysis by level of experience was performed. Subgroup analyses based on participant experience level and institutional background were not performed. Future studies may further investigate whether clinician seniority influences the impact of incremental CT imaging on the decision to operate in distal radius fractures.

## Conclusion

For AO Type B distal radius fractures, the addition of CT imaging to plain and post-reduction radiographs had minimal impact on the decision to operate. In contrast, for AO Type C fractures, the addition of 2D CT significantly influenced the decision to operate toward surgical management. However, the subsequent addition of 3D CT reconstruction did not produce a further significant change in the decision to operate.

These findings suggest that 2D CT provides clinically meaningful additional information in complex complete articular distal radius fractures, whereas 3D CT may be more valuable for preoperative planning rather than for determining the initial indication for surgery.

## Data Availability

The datasets generated and/or analyzed during the current study are available from the corresponding author on reasonable request.
